# Vitamin D and Alzheimer's Disease: Neurocognition to Therapeutics

**DOI:** 10.1155/2015/192747

**Published:** 2015-08-17

**Authors:** Anindita Banerjee, Vineet Kumar Khemka, Anirban Ganguly, Debashree Roy, Upasana Ganguly, Sasanka Chakrabarti

**Affiliations:** ^1^Department of Biochemistry, ICARE Institute of Medical Sciences and Research, Haldia 721645, India; ^2^Department of Biochemistry, Institute of Post Graduate Medical Education and Research, Kolkata 700020, India

## Abstract

Alzheimer's disease (AD), the major cause of dementia worldwide, is characterized by progressive loss of memory and cognition. The sporadic form of AD accounts for nearly 90% of the patients developing this disease. The last century has witnessed significant research to identify various mechanisms and risk factors contributing to the complex etiopathogenesis of AD by analyzing postmortem AD brains and experimenting with animal and cell culture based models. However, the treatment strategies, as of now, are only symptomatic. Accumulating evidences suggested a significant association between vitamin D deficiency, dementia, and AD. This review encompasses the beneficial role of vitamin D in neurocognition and optimal brain health along with epidemiological evidence of the high prevalence of hypovitaminosis D among aged and AD population. Moreover, disrupted signaling, altered utilization of vitamin D, and polymorphisms of several related genes including vitamin D receptor (VDR) also predispose to AD or AD-like neurodegeneration. This review explores the relationship between this gene-environmental influence and long term vitamin D deficiency as a risk factor for development of sporadic AD along with the role and rationale of therapeutic trials with vitamin D. It is, therefore, urgently warranted to further establish the role of this potentially neuroprotective vitamin in preventing and halting progressive neurodegeneration in AD patients.

## 1. Introduction

Alzheimer's disease (AD) is the most common form of dementia in the aging population. Currently 37 million people around the globe have dementia and the number is expected to double every 20 years [1, 2]. AD and AD related dementia (ADRD) are a global health problem [3]. AD is clinically characterized by progressive deficits of memory and other cognitive functions leading to complete incapacity and death within 3–9 years of diagnosis [4]. Pathological hallmarks of AD include histopathological changes induced by the extracellular deposition of amyloid *β* peptides forming senile plaques (SP) and intracellular neurofibrillary tangle (NFT) of hyperphosphorylated tau proteins in the brain [5]. Recent studies have identified that low serum concentrations of vitamin D can substantially increase the risk of AD [6]. In addition to modulating neurite growth, proliferation, differentiation, and calcium signaling, vitamin D has also been implicated in neuroprotection and may alter neurotransmission and synaptic plasticity [7]. Brain imaging studies have linked hypovitaminosis D to dysfunction of the frontal-subcortical neuronal circuits [8]. Vitamin D deficiency has also been linked with increasing hypertension, hyperlipidemia, myocardial infarction, and stroke which are also risk factors for AD [9]. This review will focus primarily on the complex underlying mechanisms that promote vitamin D deficiency as a major contributory factor in the progression of sporadic AD and analyze its potential as a possible therapeutic target.

## 2. Pathogenesis of AD

**Figure 1:**
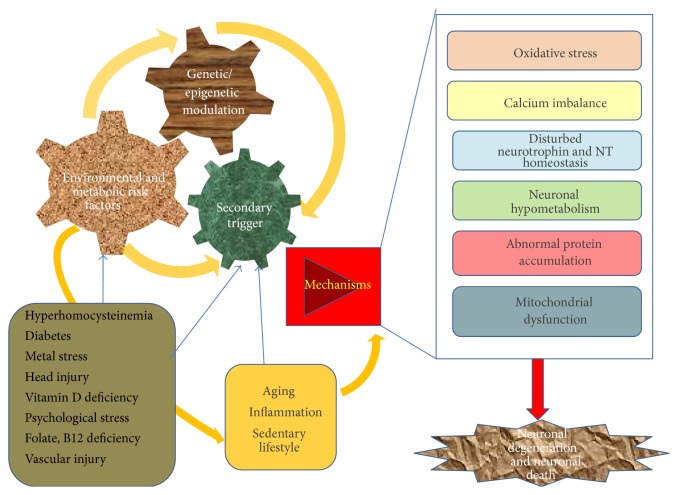
Risk factors and pathogenic mechanisms in the aetiopathogenesis of sporadic Alzheimer's disease (AD).

AD is a multifactorial disease and the mechanisms underlying its pathogenesis are complex. Several postmortem evidences, studies in transgenic animal models, and cell-based models (cell lines and primary cortical neurons) have improved our understanding of the pathogenesis of AD [10–14]. These studies have implicated amyloid *β* (A*β*) accumulation, hyperphosphorylated tau, oxidative stress, metal dysregulation, mitochondrial dysfunction, and inflammatory response as major interconnecting networks leading to neuronal and synaptic degeneration [15–18]. Alterations in the amyloid metabolic cascade constitute an important hypothesis in AD, though none of the theories alone is sufficient to decipher the biochemical and pathological complexities that result in disease progression [19]. Cortical plaques in AD brain primarily contain A*β* protein which is produced from its parent amyloid precursor protein (APP) through sequential hydrolysis by *β* and *γ*-secretases [20]. The major species of A*β* are A*β*-40 and A*β*-42 peptides and the latter is predominant in neuritic plaques and has a higher propensity to aggregate and form the characteristic toxic amyloid fibrils in AD [21, 22]. In spite of evidences in support of “amyloid cascade hypothesis” and “tauopathy,” it is still unclear how these events are triggered in the aging brain and how they contribute to the complexity and the heterogeneity of AD. Moreover, it has been established that the etiology of sporadic AD involves multiple gene-environment interactions as well as epigenetic mechanisms (as shown in Figure 1) working in the backdrop of aging brain [23]. An interesting hypothesis in this regard is the Latent Early-life Associated Regulation (LEARn) model which proposes that exposure to various environmental risk factors (heavy metals or nutritional deficiency) in the early developmental life can bring about epigenetic modifications of AD related genes (first hit) which remain latent for many years until a second hit (aging, elevated proinflammatory cytokines, and diet) results in sustained alterations in these genes promoting disease progression [24].

## 3. Vitamin D Metabolism

Although vitamin D (calciferol) was discovered in the early 20th century as a vitamin, it is now recognized as a prohormone [25, 26]. Calciferols are a group of fat soluble secosterols broadly divided into two major forms: ergocalciferol (vitamin D_2_) and cholecalciferol (vitamin D_3_) [27]. While vitamin D_2_ is largely found in food, vitamin D_3_ is synthesized in the human skin by a photochemical reaction (ultraviolet B 297–315 nm) from 7-dehydrocholesterol [28] and is also consumed in the diet. Vitamin D, in either D_2_ or D_3_ form, is considered biologically inert until it undergoes two enzymatic hydroxylation reactions. First, vitamin D binds carrier proteins in the skin (particularly the vitamin D binding protein or DBPs) and is transported to the liver [29] where it is enzymatically hydroxylated by vitamin D-25-hydroxylase (CYP2R) on C-25 thereby generating 25(OH)D or calcidiol. A second hydroxylation reaction in the kidney by 25(OH) D-1-OHase (CYP27B1) hydroxylates 25(OH)D at C-1*α* position and converts it to the biologically active form 1,25-dihydroxyvitamin D (1,25(OH)_2_D) or calcitriol [30]. 1,25(OH)_2_D concentration in the blood is regulated by a feedback mechanism and by the induction of parathyroid hormone, Ca2+, and various cytokines. Recent studies have shown that in addition to renal cells, various other cells (keratinocytes, monocytes, macrophages, osteoblasts, prostate, and colon cells) are capable of carrying out the second hydroxylation reaction [31–33]. Circulating 25(OH) vitamin D crosses the blood-brain barrier and enters neuronal and glial cells to be converted to 1,25(OH)_2_D [34, 35]. CYP27B1 has also been detected in developing human fetal brain [36]. Recent data has shown that the central nervous system can locally perform bioactivation of vitamin D prohormone and the presence of 1 *α*-hydroxylase in human brain. In this regard, it is worth mentioning that microglial cells in culture produce 1,25(OH)_2_D from its precursor [37]. The pattern of distribution of CYP27B1 in human and rat brains is more or less consistent. Previous studies have identified the presence of another key enzyme, CYP24A1, which is involved in vitamin D catabolism in the human brain and also shown that CYP24A1 mRNA has been induced by the treatment of glial cells with 1,25 OH vitamin D [38]. Thus, 1,25(OH)_2_D is produced in various organs and cells, functions in an autocrine pathway, and leads to its own destruction by activation of CYP24A1 which hydroxylates and oxidizes it to form the inactive calcitroic acid [30].

Recent evidences link serum vitamin D deficiency to cognitive impairment and dementia [39, 40]. Vitamin D receptors are widely expressed in the brain [41]. Expression of the active form of vitamin D regulates neurotrophin levels and the survival of neural cells [42]. In vitro studies show that vitamin D can stimulate the clearance of amyloid plaques by inducing phagocytosis by macrophages and also reduces the amyloid-induced cytotoxicity, apoptosis, and inflammatory responses in primary cortical neurons [43]. Thus, supplementation with vitamin D may ameliorate the cognitive deficits in the elderly people and control neuronal health and homeostasis.

## 4. Vitamin D and Neuroprotection

The effects of vitamin D are exerted through its nuclear hormone receptor, vitamin D receptor (VDR), or its membrane receptor, membrane-associated, rapid-response, steroid-binding protein (1,25 MARRS) [44]. Genomic and nongenomic actions of vitamin D are mediated by nuclear and membrane receptors, respectively, having identical receptor VDR in both locations. Classical 1,25(OH)_2_D genomic signaling, which is conducted through the VDR, has structural similarities with the nuclear steroid receptor family. VDR gene is present on chromosome 12q13.11. In 1992, with the help of in situ hybridization studies, it was proved for the first time that VDR is expressed in the human brain [45]. Expression of VDR mRNA in postmortem brains of patients with Alzheimer's or Huntington's disease was identified using radiolabeled cDNA probes. Human neuroblastoma cell line was also shown to express VDR [41, 46]. The presence of VDR in the astrocytes is indicated by its presence in glial fibrillary acidic protein (GFAP) stained cells in primary rat hippocampal cultures [41, 47]. Secondary oligodendrocyte cultures and glial cell-line studies verified this statement [48]. The potential role of vitamin D in cellular development and differentiation is suggested by its expression in developing brain [41, 49]. Bartoccini and colleagues have shown the rapidly partitioning of VDR into the lipid rich microdomains within the nuclear membrane in developing hippocampal neurons. These microdomains have similar characteristics of those found in the plasma membrane [50]. Almeras et al. have shown that adult brain functioning can be affected by vitamin D deficiency during development, in a rat model [51]. Vitamin D regulates transcription of various genes, by binding to nucleus, forming a heterodimer with RXR and subsequently translocating to vitamin D response element in the DNA [52–55]. Recent report suggests that VDR is widely expressed throughout the CNS with the highest expression in the hippocampus, hypothalamus, thalamus, cortex, and subcortex and substantia nigra, the areas essential for cognition.

### 4.1. Vitamin D and Regulation of NGF and Neurotransmitters

1,25-Dihydroxyvitamin D [1,25(OH)_2_D] plays a pivotal role in neuronal differentiation and maturation via control of the synthesis of neurotrophic agents such as nerve growth factor (NGF) and glial cell-line-derived neurotrophic factor (GDNF), neurotrophin 3, and the synthesis of low-affinity p75 NTR receptors [56]. Nerve growth factor is important for the growth, maintenance, and survival of certain target neurons and also has been implicated in maintaining and regulating the normal functioning of the septohippocampal pathway, which is involved in learning and memory. It has been noted that mature NGF levels are substantially decreased in the forebrain of aged animals and patients with AD. In vitro studies using neuronal PC12 cells have successfully shown that APP gene expression is modulated by NGF, and an increase in APP expression is noted upon its withdrawal [57]. Calcitriol and vitamin D analogs were reported to enhance NGF induction by increasing AP-1 binding activity in the NGF promoter, in mouse fibroblasts [58]. The genetic expression of numerous neurotransmitters in the brain, including acetylcholine, dopamine, serotonin, and *γ*-aminobutyric acid is regulated by 1,25(OH)D and that is notably in the hippocampus [59–61].

### 4.2. Vitamin D and Calcium Homeostasis

It has been well known by the virtue of previous studies that aging brain and also Alzheimer's disease show calcium dysregulation, thus coining the term “Ca^2+^ hypothesis of brain aging and dementia” [62]. The L-type voltage sensitive Ca^2+^ channel, one of the most important proteins in calcium metabolism, aging, and neurodegeneration is worth mentioning in this context as it is reported that vitamin D regulates intraneuronal calcium homoeostasis via the regulation of these channels, including those targeted by A*β* [63, 64]. Moreover, rapid increase in LVSCC-A1C expression in response to VDR silencing was found in a study by Gezen-Ak et al. which indicates that chronic inefficiency in vitamin D utilization in brain renders the neurons vulnerable to neurodegeneration [62, 65]. Vitamin D treatment leads to downregulation of LVSCC expression, L-type currents, and channel density in the plasma membranes of the hippocampal neurons which is the possible explanation for the protection of the neurons from calcium excitotoxicity [63].

### 4.3. Anti-Inflammatory Role of Vitamin D

The potent immune-modulatory and anti-inflammatory action of vitamin D has long been elicited. Age-related inflammatory changes in the hippocampus may be reversed by vitamin D as shown in mice models [46]. Suppression of proinflammatory cytokines in the brain may be the probable mechanism of action for this neuroprotection [66, 67]. Lipopolysaccharide-induced levels of mRNA encoding macrophage colony-stimulating factor (M-CSF) and tumor necrosis factor *α* (TNF-*α*) in cultured astrocytes are partially reduced by vitamin D treatment, as shown in few studies [61]. Alteration of neurotransmitter synthesis by proinflammatory cytokines, such as IL-1*β* and IL-6, can have detrimental effect on behaviour and conditioned learning [67]. Calcitriol and its analogs have also been shown to be associated with the regulation of prostaglandin metabolism and selective inhibition of COX-2 activity [68, 69].

### 4.4. Vitamin D and Amyloid Beta Metabolism

Nevertheless, it is worth mentioning that vitamin D also regulates the APP and amyloid beta metabolic aspects. The promising role of 1,25(OH)_2_D in recovering the ability of the macrophages to phagocytose soluble amyloid *β* protein came to surface in a very recent work in macrophages from patients with Alzheimer's disease [43]. Another study has shown its ability to attenuate amyloid *β* (A*β*)42 accumulation by stimulating phagocytosis of the A*β* peptide [70] and enhancing brain-to-blood A*β* efflux across BBB [71], resulting in decreased number of amyloid plaques [72]. VDR interacts with SMAD 3, which is involved in APP processing through TGF-beta signaling, as a transcription factor [73, 74].

### 4.5. Vitamin D and Brain Oxidative Stress

Finally, vitamin D was shown to exhibit neuroprotective properties against glutamate toxicity. Cultured rat cortical neurons were protected from acute glutamate exposure by vitamin D treatment, through the upregulation of VDR expression and antioxidant effects [75, 76]. Various reports show that vitamin D exerts its protecting effects against free radicals generated by reactive species of oxygen and nitric oxide, inhibits the synthesis of inducible nitric oxide synthase, and regulates the activity of the gamma glutamyl transpeptidase, which is a key enzyme involved in the metabolism of glutathione [76–78]. Vitamin D is found in increasing glutathione levels in mesencephalic dopaminergic neurons even after treatment with various neurotoxins or inhibitors of glutathione synthesis [79]. 1,25(OH)_2_D also protects from cerebral endothelial dysfunction by its inhibitory effects on ROS production and NF-*κ*B activation. Similarly, the bEnd 3 cells (mouse brain endothelial cell line) when treated with 1,25(OH)_2_D were found to be protected from hypoxic/oxidative insults. The inhibitory action of vitamin D on I*κ*B phosphorylation and P65 translocation to the nucleus accounts for this protective effect [80].

Vitamin D supplementation also enhances brain energy homeostasis and protein phosphatase 2A (PP2A) activity and modulates the redox state and thus reduces age-related tau hyperphosphorylation and cognitive impairment [81]. Gene expression in the whole brain and protein expression in the prefrontal cortex and hippocampus of adult vitamin D-deficient rats has been explored with the help of gene array and proteomics analysis. Expression of 74 genes and 36 proteins involved in diverse functions such as cytoskeleton maintenance, calcium homeostasis, synaptic plasticity and neurotransmission, oxidative phosphorylation, redox balance, protein transport, chaperoning, cell cycle control, and posttranslational modifications were significantly altered in vitamin D deficiency [51, 82, 83]. Moreover, vitamin D_2_ enriched button mushroom (*Agaricus bisporus*) is found to improve memory in both wild type and APPswe/PSIdE9 transgenic mice. It is worth mentioning that some very recent studies have identified the vitamin D binding protein (DBP), a prealbumin, interacting with A*β*. DBP has been shown to inhibit oligomerization of A*β* in vitro as well as preventing A*β* induced hippocampal synaptic loss and resulting memory impairment [84].

**Figure 2:**
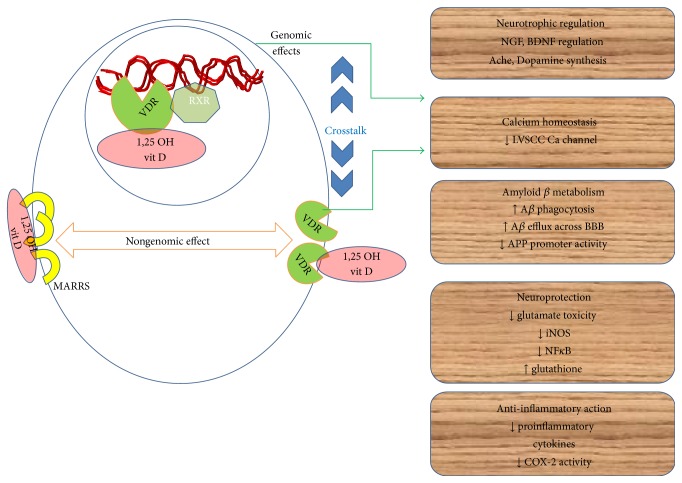
Mechanisms of 1,25 OH vitamin D mediated multitargeted neuroprotection in AD. VDR: vitamin D receptor, RXR: retinoid X receptor, MARRS: membrane associated rapid response receptors, and LVSCC: L voltage sensitive calcium channel.

Multiple pathogenic mechanisms implicated in the pathogenesis of sporadic AD and multifaceted neuroprotective role of 1,25-dihydroxyvitamin D are summed up above (Figure 2). In addition to the above mentioned mechanisms, the protective role of vitamin D in the cardiovascular system is exerted through cardiac remodeling, endothelial response regulation to injury, and blood coagulation, ultimately improving the CNS vascular homeostasis. Thus, in this manner vitamin D indirectly protects the brain form cerebrovascular risk factors of AD [85].

## 5. Evidences Linking Vitamin D Deficiency, Neurocognition and AD

First, Llewellyn et al. showed an increased risk of losing points on Mini-Mental State Examination (MMSE) in 6 years among 175 older adults with baseline 25(OH)D 10 ng/mL compared to 157 subjects with 25(OH)D 30 ng/mL [86, 87]. Second, Slinin et al. followed up the association between lower 25(OH)D levels and cognitive decline among aged population (>65 years) for more than 4 years [87, 88]. A meta-analysis by Etgen et al. highlighted an increased risk of cognitive impairment in patients with vitamin D deficiency [89]. Balion et al. compared mean MMSE scores with levels of 25(OH)D, where he showed a higher average MMSE score in those participants with higher 25(OH)D concentrations [90]. Further, in the In CHIANTI study consisting of 858 adults, cognitive decline were associated with low concentrations of vitamin D, when observed over a period of 6 years [86]. Nevertheless, the intriguing query remains the same: does hypovitaminosis D contribute to cognitive decline or is it the other way round? Deficient sun exposure and feeding difficulties with subsequent fewer intakes of vitamin D-rich foods are predisposed by AD disease process itself. But this is contradictory to the fact that in cases with mild AD, where major functional disabilities have not yet started, the association between low 25(OH)D concentrations and AD still persists [91]. Importantly, longitudinal prospective studies have established the temporal relation between hypovitaminosis D and cognitive disorders, where older individuals with lower vitamin D levels had a significantly higher risk of global cognitive decline and executive dysfunction compared to those with normal/higher vitamin D levels [86, 92, 93]. Moreover, to add to the above mentioned fact, studies have reported that low vitamin D was associated with an increased risk of AD [94]. More than 50% of the prospective studies showed an elevated risk of cognitive impairment after 4–7 years of follow up, in participants with lower 25(OH)D levels, when compared with participants with higher 25(OH)D levels [95]. Other cross-sectional studies have showed increased incidence of vitamin D deficiency in AD patients [87, 90].

### 5.1. VDR and AD

Genome-wide analyses, transcriptomics, and proteomics approaches have pointed the role of various genes in increasing AD liability, for example, inflammatory genes (IL1, IL6), oxidative stress (NOS), VDR, cathepsin, ubiquilin, COMT, and AChE [96]. With VDR being the major mediator of vitamin D actions, recent genome-wide association studies have focused on finding the role of VDR polymorphism in late onset Alzheimer's disease (LOAD) susceptibility [97]. A decreased level of VDR mRNA has been reported in hippocampal region by analyzing postmortem AD brain [45].

Alteration in receptors VDR and 1,25-MARRS (membrane associated, rapid response steroid-binding), genes related to the action, and metabolism of vitamin D result in inefficient utilization of vitamin D, making neurons vulnerable to neurodegenerative changes [64, 65, 97–100]. Association between AD and polymorphisms of VDR and megalin strongly supports this notion and, therefore, explains the neurotoxic effects of VDR and 1,25-MARRS suppression [62, 65, 97, 99–101].

### 5.2. VDR Silencing

To study VDR silencing, the VDR knockout (VDR−/−) mouse model of deranged vitamin D-VDR signaling and poor utilization of vitamin D in the CNS is widely accepted. Several phenotypes of the VDR−/− mouse have been identified. University of Tokyo (Japan) initially generated VDR KO mice which show the signs of shortened lifespan and premature aging. Thus, it is counted as an impressive model of VD and VDR deficiency as seen in accelerated brain aging. Cognitive and behavioral deficits and calcium dysregulation in brain are also found to affect VDR KO mice [102–104].

VDR and NGF level are found to be suppressed as a result of amyloid beta toxicity [62]. This reinforces the idea that vitamin D suppression is the reason behind the decreased NGF levels in A*β* induced neurotoxicity. It can be presumed that A*β* induces VDR protein degradation triggering some unknown mechanisms. Inadequate vitamin D levels in AD patients, along with VDR protein depletion, can precipitate a cascade of critical phenomenon. All these information point to the fact that even sufficient levels of vitamin D are not enough for normal functioning, as A*β* can create hindrance by downregulating VDR [64]. VDR silencing can also upregulate LVSCC Ca channels. In a study by Gezen-Ak et al., VDR siRNA-treated cortical neurons showed upregulated LVSCC-A1C mRNA levels after both 12 and 24 hours of treatment, compared to the control. Protein levels followed the same trend [62].

### 5.3. VDR Polymorphism and AD

The critical steps in the control of gene expression by VDR include ligand binding, with retinoid X receptor (RXR) heterodimerization and binding of the heterodimer to VDR response elements (VDREs). Thus genetic polymorphisms of VDR gene can lead to defects in important gene activations. Polymorphisms can take place in the noncoding parts of the genes or introns whereby they are not translated into the protein. However, polymorphisms within the regulatory regions can affect the gene expression. Polymorphisms within the 5′ promoter region of VDR gene affects mRNA expression patterns and levels while that within the 3′ untranslated region (UTR) affects mRNA stability [105]. The existence of several single nucleotide polymorphisms (SNPs) within the VDR gene has been described using restriction enzymes which include Tru9I, TaqI, BsmI, EcoRV, ApaI, and FokI [106–109]. Using sequencing approaches, Cdx2 polymorphism was found [110]. Some of these common VDR polymorphisms have been linked to AD. Gezen-Ak et al. reported the association of ApaI polymorphism and not TaqI polymorphism in late onset AD in the studied Turkish population thereby indicating that polymorphism within the ligand binding site of VDR gene increases the risk of AD progression [100]. However, Lehmann et al. and Lee et al. demonstrated the existence of possible links between ApaI and TaqI with the risk of AD [111, 112]. Wang et al. reported the association between Cdx2 and lower VDR promoter activity thereby increasing susceptibility to AD [113]. Thus, VDR polymorphism may decrease the affinity of vitamin D to VDR and affect the expression of neurotrophins. This can lead to neuronal aging and neurodegeneration in association with other genetic and environmental factors.

## 6. Is Vitamin D Essential in AD Therapeutics?

Despite several experimental in vivo or in vitro models of AD explaining its molecular pathogenesis which have led to different types of drug treatment strategies and tests in animal and cell-based models and in clinical trials, the treatment of AD in general is terribly inadequate at present. The disease progresses relentlessly with devastating failure of memory and cognition till the patient succumbs to the illness usually by 5–9 years after the diagnosis. A major focus of the drug treatment for AD is to improve cognitive abilities, such as memory and thinking, and slow the progression of these symptoms. Four drugs are currently approved by the US Food and Drug Administration (FDA) for treating cognitive symptoms of AD. Three of them (galantamine, rivastigmine, and donepezil) act as anticholinesterase agents while memantine acts by preventing excitatory neuronal damage [114].

In general, several strategies have been evolved to halt the progression of the disease such as increasing the clearance of amyloid beta peptide from the brain by active or passive immunization against the peptide or promoting enzymatic degradation of the peptide, diminishing the synthesis of A*β*-42 by inhibition of *β*-secretases or *γ*-secretases, activation of nonamyloidogenic processing of APP through modulation of *β*-secretase action, preventing the aggregation and fibrillization of A*β*-42, inhibiting tau phosphorylation, antibodies against A*β*, and reducing inflammation or oxidative stress or excitotoxicity [114–116]. Apparently, either they are still in the trial period or some trials have shown nonpromising results.

The complexity of the mechanisms involved in AD has prompted the researchers to develop compounds that could simultaneously interact with several potential targets (multitarget directed ligand design) [117]. Variety of compounds with dual or multiple target specificities are in development. As vitamin D interacts with different mechanisms, therefore, it is a multitargeted therapeutic option for prevention and protection from cognitive decline and AD. The protective role of vitamin D in AD has been clearly established as discussed above. Now this is an important point when considering randomized controlled trials since it seems almost impossible to get a trial approved to examine the effectiveness of vitamin D alone in AD and/or ADRD patients after having removed standard therapies. A 7-year follow-up study by Annweiler et al. confirms that higher vitamin D dietary intake was associated with lower risk of developing AD among older women [94]. The AD-IDEA trial, a randomized placebo-controlled trial, was the first of such trials, on the effectiveness of vitamin D in ADRD patients [118].

The trial of vitamin D alone or in combination with other agents/anti-AD drugs have shown positive results in some recent works. Fiala and Mizwicki have shown that combined use of vitamin D_3_ and DHA (Docosahexaenoic acid) is an emerging novel strategy to enhance direct and immune protection of neurons against brain amyloidosis and other brain insults [119]. As vitamin D targets various pathological processes of ADRDs, it may increase the effectiveness of standard antidementia treatments or account at least partially for the resistance to these treatments. In support of this fact, a recent 6-month trial study by Annweiler et al. has shown that the combination of memantine and vitamin D was superior to either memantine or vitamin D alone in halting the cognitive decline amongst participants with AD as evidenced by improved MMSE score [120].

In contrast, one randomized control by Stein et al. showed that neither cognition nor disability changed significantly after high-dose vitamin D in mild to moderately severe AD cases [121]. Another study also found no improvement of cognition in an elderly nursing home residents after 4 weeks of oral vitamin D_2_ supplementation [122]. These studies, by Stein etal. or by Przybelski et al. have shown no benefits of high-dose vitamin D_2_ supplementation on cognition [121, 122]. However, there have been some methodological limitations that affected their conclusions. For instance, both studies monitored the role of vitamin D_2_ supplements which are generally less efficient than vitamin D_3_ for repletion, and duration of the follow-up that did not exceed 16 weeks, while the effects of vitamin D can be observed after a longer period [123]. Additionally, none of these studies assessed executive functions or episodic memory as outcome measures, although serum 25OH vitamin D concentrations are associated with these domain-specific cognitive functions [124]. The arguments against vitamin D supplementation are based on the small number of clinical trials. Further well-conducted randomized clinical trials (RCTs) to test the effectiveness of vitamin D supplements against placebo in patients with AD are essentially needed at this time.

## 7. Conclusion and Future Directions

The pathophysiology of AD involves the accelerated aging of neurons leading to alteration in neuronal metabolism and stability. A link between premature aging, diet, and nutrition is proposed with nutrigenomic research uncovering possible mechanisms such as epigenetic modifications that demonstrate the interaction between genes and environment disturbances and imbalances occurring in a variety of mechanisms. It surprises that, in spite of the wealth of knowledge that exists regarding AD, only a handful of options are available currently for its management. The disease process is also complex in its own ways. Symptomatic treatment is the best part of the management currently. However, exciting and incredible leaps have taken place in developing disease modifying approaches. The failed antiamyloid and antioxidant drug trials confirms that the understanding of the AD pathology still needs to be dissected out in every aspect. Detailed exploration of the link between several metabolic and endocrine etiological factors, gene-environment interactions and their influence on MCI and subsequent AD progression are of prime focus now and treatment strategies should be looked up very carefully. Inappropriate timing and the long latency period of AD adds another dimension in its complexity wherein the principal mechanisms change with the time course and progression of the disease. Antioxidant therapy may be helpful in the early stages of the disease but not when sufficient damage has already been done. Opposite is the case with memantine which is useful in moderate to severe stages, but not in early AD. Multitargeted neuroprotective action of vitamin D makes it a lucrative candidate for the prevention as well as treatment strategy in AD and ADRDs. A recent task force on vitamin D and neurocognition has analysed all major studies and enabled international experts to reach to the agreement that hypovitaminosis D and the inefficient utilization of vitamin D increase the risk of cognitive decline/ADRDs in older adults and may alter the clinical presentation of the disease, particularly as a sequel of accompanying morbidities [90]. However, at present, hypovitaminosis D should not be used as a diagnostic or a prognostic biomarker of cognitive decline/ADRDs due to lack of specificity and insufficient evidence. The experts recommended measurement of serum 25(OH)D because of the high prevalence of hypovitaminosis D in this population and supplementation, if necessary. However, it is worth noting that a very recent study has identified vitamin D binding protein to be a potential blood biomarker for the diagnosis of AD [125]. However, further verification with larger epidemiological and molecular evidences is eagerly awaited before targeting DBP as a possible therapeutic modulation in AD.

At this time, further laboratory experiments, prospective studies and large trials are essential to clarify the mechanisms through which vitamin D benefits the brain. A stronger focus on the role of vitamin D-related genetic variance (e.g., in the genes encoding VDR, *α*-hydroxylase, or VDBP) in humans is also necessary in order to understand the prevalence and therapeutic response. In parallel, conditional and brain-specific VDR mutations have to be identified to assess their neurophenotypes. Acute and chronic vitamin D treatment or depletion should also be carried out in order to best interpret neurocognitive and behavioral abnormalities associated with vitamin D deprivation. Further, animal-based studies and drug trials need to be conducted to determine the threshold concentration of vitamin D to prevent neurodegeneration, so that the correct dose of supplementation could be determined.
